# Real-world data of first-line treatment with pembrolizumab for NSCLC with high PD-L1 expression in elderly patients: a subgroup analysis of HOT/NJLCG2001

**DOI:** 10.1093/jjco/hyae168

**Published:** 2024-12-04

**Authors:** Kazunari Tateishi, Hidenori Mizugaki, Yasuyuki Ikezawa, Ryo Morita, Keiki Yokoo, Toshiyuki Sumi, Mari Aso, Hajime Kikuchi, Atsushi Nakamura, Motoki Sekikawa, Fumiaki Yoshiike, Yasuo Kitamura, Nozomu Kimura, Tsutomu Hachiya, Kyoji Tsurumi, Toshihiko Agatsuma, Furuta Megumi, Keiichi Nakamura, Daisuke Jingu, Hiroshi Yamamoto, Makoto Kosaka, Hiroshi Yokouchi

**Affiliations:** First Department of Internal Medicine, Shinshu University School of Medicine, Matsumoto, Japan; Department of Respiratory Medicine, NHO Hokkaido Cancer Center, Sapporo, Japan; Department of Advanced Medical Development, The Cancer Institute Hospital of Japanese Foundation for Cancer Research, Tokyo, Japan; Department of Respiratory Medicine, Oji General Hospital, Tomakomai, Japan; Department of Respiratory Medicine, Faculty of Medicine, Hokkaido University, Sapporo, Japan; Department of Respiratory Medicine, Akita Kousei Medical Center, Akita, Japan; Department of Respiratory Medicine, Teine Keijinkai Hospital, Sapporo, Japan; Department of Respiratory Medicine, Hakodate Goryoukaku Hospital, Hakodate, Japan; Department of Respiratory Medicine, Yamagata Prefectural Central Hospital, Yamagata, Japan; Department of Respiratory Medicine, Obihiro-Kousei General Hospital, Obihiro, Japan; Department of Pulmonary Medicine, Sendai Kousei Hospital, Sendai, Japan; Department of Respiratory Medicine, Steel Memorial Muroran Hospital, Muroran, Japan; Department of Respiratory Medicine, Nagano Municipal Hospital, Nagano, Japan; Department of Respiratory Medicine, Kushiro City General Hospital, Kushiro, Japan; Department of Respiratory Medicine, Tohoku University Graduate School of Medicine, Sendai, Japan; Department of Respiratory Medicine, Japanese Red Cross Suwa Hospital, Suwa, Japan; Department of Respiratory Medicine, Miyagi Cancer Center, Natori, Japan; Department of Respiratory Medicine, Shinshu Ueda Medical Center, Ueda, Japan; Department of Respiratory Medicine, Faculty of Medicine, Hokkaido University, Sapporo, Japan; Department of Respiratory Medicine, National Hospital Organization Asahikawa Medical Center, Asahikawa, Japan; Department of Respiratory Medicine, Saka General Hospital, Shiogama, Japan; Department of Respiratory Medicine, Iida Municipal Hospital, Iida, Japan; Center of Infectious Diseases, Nagano Prefectural Shinshu Medical Center, Suzaka, Japan; Department of Respiratory Medicine, NHO Hokkaido Cancer Center, Sapporo, Japan

**Keywords:** chemotherapy, elderly patients, non-small cell lung cancer, lung cancer, PD-L1

## Abstract

**Background:**

In the first-line treatment of elderly patients with advanced-stage non-small cell lung cancer (NSCLC) with high programmed death-ligand 1 (PD-L1) expression (tumor proportion score ≥ 50%), this study aimed to determine whether pembrolizumab monotherapy (MONO) or pembrolizumab plus platinum-based chemotherapy (COMB) should be selected.

**Methods:**

We performed a retrospective multicenter study (sub-analysis of the HOT/NJLCG2001 trial) of 299 patients with NSCLC with high PD-L1 expression who received MONO or COMB as the first-line treatment between December 2018 and January 2020. We selected patients aged 75 years and older and assessed the clinical efficacy and toxicity.

**Results:**

In total, 81 (median age: 79 years) and 19 (median age: 76 years) patients received MONO and COMB, respectively. Twenty patients with a performance status (PS) score of 2–3 were enrolled in the MONO group. The median progression-free survival (PFS) was 7.8 and 8.9 months in the MONO and COMB groups, respectively. The median overall survival (OS) was 14.6 and 20.3 months, and the 2-year survival rates were 38.8 and 49.9%, respectively. Furthermore, 29.6% and 26.3% of patients discontinued treatment due to adverse events, respectively. In MONO, patients with PS 0–1 had a longer PFS (10.5 months) and OS (21.7 months) than those with PS 2–3 (0.7 and 1.6 months, respectively).

**Conclusion:**

Some elderly patients with NSCLC and high PD-L1 expression might benefit from COMB; however, MONO is considered the preferred treatment. MONO may not be an effective or feasible treatment for patients with PS 2–3, even with high PD-L1 expression.

## Teaser

In this subgroup analysis of the HOT/NJLCG2001 trial, pembrolizumab monotherapy was the preferred treatment for elderly patients with advanced-stage non-small cell lung cancer with high PD-L1 expression.

## Introduction

Immune checkpoint inhibitors (ICIs) play an important role in the first-line treatment of advanced non-small cell lung cancer (NSCLC) without driver gene mutations. When compared with platinum-based chemotherapy, patients with high programmed death-ligand 1 (PD-L1) expression (PD-L1 tumor proportion score [TPS] ≥50%) who received first-line treatment with pembrolizumab [a programmed cell death protein-1 (PD-1) inhibitor and humanized monoclonal antibody] as either monotherapy (MONO) or combined with chemotherapy (COMB) demonstrated improved progression-free survival (PFS) and overall survival (OS) (1–5). In general, the incidence of treatment-related adverse events (AEs) was lower in the patients treated with MONO than in those treated with COMB; however, no phase III trials have directly compared MONO and COMB, and whether MONO or COMB should be administered to NSCLC patients with high PD-L1 expression is open to investigation.

We previously conducted a retrospective multicenter observational trial in the Hokkaido Lung Cancer Clinical Study Group (HOT) and North Japan Lung Cancer Study Group (NJLCG) in 2001 (6,7). Although COMB may have restricted patient selection, it could be beneficial for the first-line treatment of NSCLC with high PD-L1 expression.

NSCLC is often detected in elderly populations. Based on the Surveillance, Epidemiology and End Results (SEER) database, as of 2017–2021, 36.6% of new lung cancer cases were diagnosed in patients aged 75 years and older (8). In elderly patients, treatment regimens should be selected based on the AEs and therapeutic effects. In the KEYNOTE-024 trial, which included patients with high PD-L1 expression, 45 patients (14.8%) were aged 75 years and older (1). In the KEYNOTE-189 and KEYNOTE-407 trials, which included patients with any PD-L1 status, 57 patients (9.3%) and 65 patients (11.6%) were aged 75 years and older, respectively (2,3,9,10). However, the treatment efficacy and AEs in elderly patients in these trials are not clear. Although some retrospective studies have shown the therapeutic efficacy and AEs associated with MONO in elderly patients with NSCLC and high PD-L1 expression (11), few studies have compared it with COMB (12,13).

In this study, we used real-world data from the HOT/NJLCG2001 cohort to explore the treatment selection, treatment efficacy and AEs in patients aged 75 years and older with NSCLC and high PD-L1 expression.

## Materials and methods

### Study design

We previously conducted a retrospective multicenter observational trial, HOT/NJLCG2001 (UMIN000040223) (6). The present study was a subgroup analysis of the HOT/NJLCG2001 trial. The patient selection consort diagrams are presented in Supplement S1. Eligible patients had previously untreated advanced NSCLC with a PD-L1 TPS of ≥50% and no sensitizing *EGFR*, *ALK* or *ROS-1* alterations; additionally, they received MONO or COMB as the first-line treatment between December 2018 and January 2020. Patients 75 years or older were selected from the initial pool of 299 enrolled patients, with a total of 100 patients chosen for the study. The efficacies and toxicities of MONO and COMB were evaluated. We reviewed the medical records of 34 Japanese institutions. This study was approved by the institutional review boards of all the institutions, which waived the need for informed consent owing to the anonymous nature of the data. The data cutoff date for this study was August 2022.

### Data collection

We assessed the patient characteristics, therapeutic regimens, treatment periods, PFS, OS, 2-year survival rate and AEs, and recorded the age, sex, smoking status, histology, clinical stage, PD-L1 status and Eastern Cooperative Oncology Group (ECOG) performance status (PS) score at the start of the initial treatment. We also assessed PD-L1 expression using the PD-L1 IHC 22C3 pharmDx assay (Agilent) and recorded the therapeutic regimens (MONO or COMB) and types of chemotherapy (carboplatin + pemetrexed, cisplatin + pemetrexed, carboplatin + nab-paclitaxel and carboplatin + paclitaxel). We extracted AE types of grade 3 or higher and AEs leading to treatment discontinuation using the common terminology criteria for AEs (CTCAE ver. 5.0) (14). Tumor response was measured using the Response Evaluation Criteria in Solid Tumors version 1.1 (RECIST 1.1) (15), and assessments were performed at each participating institution.

### Statistical analyses

Details of the statistical analysis have been described previously (6,7). PFS was defined as the time interval between the initial treatment administration and disease progression or death. Patients without documented clinical or radiographic disease progression or those who were still alive were censored on the date of the last follow-up. OS was defined as the time interval between the initial treatment administration and any cause of death. PFS and OS were evaluated using the Kaplan–Meier method and compared using a two-sided log-rank test. Hazard ratios (HRs) and 95% confidence intervals (CIs) were estimated using a Cox proportional hazards regression model. Two-year survival rate was calculated for comparison with the Phase 3 trial (the KEYNOTE-024, the KEYNOTE-189 and KEYNOTE-407 trials). Multivariate analyses using Cox proportional hazards modeling were performed to measure the correlations between PFS and OS and the following factors: patient characteristics (age, sex, PS and smoking), tumor factors (clinical stage, pathological diagnosis and PD-L1 status) and pembrolizumab treatment (MONO or COMB). All *P* values were two-sided, and the threshold for statistical significance was set at *P* < 0.05. The IBM SPSS Statistics software (version 26; IBM Corp., Armonk, NY, USA) was used for all statistical analyses.

## Results

### Patient characteristics

The patient characteristics are shown in Table 1. Of the 100 patients, 81 (81%) and 19 (19%) underwent MONO and COMB, respectively. The median follow-up duration was 13.4 months (range: 0.1–44.1 months) for all patients. The median patient age was 79 years (range: 75–89 years) and 76 years (range: 75–84 years) in the MONO and COMB groups, respectively. There were 61 patients (75.3%) with a PS score of 0–1 in the MONO group and 19 patients (100%) in the COMB group.

**Table 1:** Baseline and treatment characteristics

	No. (%)						
Characteristics	All (*n* = 100)		MONO (*n* = 81)		COMB (*n* = 19)		*P* value
Age in years							0.772
Median (range)	78	(75–89)	79	(75–89)	76	(75–84)	
Sex							0.512
Male	82	(82.0)	65	(80.2)	17	(89.5)	
Female	18	(18.0)	16	(19.8)	2	(10.5)	
Smoking status							0.514
Current smoker	9	(9.0)	6	(7.4)	3	(15.8)	
Former smoker	73	(73.0)	60	(74.1)	13	(68.4)	
Never smoker	18	(18.0)	15	(18.5)	3	(15.8)	
Histology							0.104
Adenocarcinoma	53	(53.0)	47	(58.0)	6	(31.6)	
Squamous cell carcinoma	38	(38.0)	27	(33.3)	11	(47.9)	
Nonsmall cell carcinoma	7	(7.0)	5	(6.2)	2	(10.5)	
Others	2	(2.0)	2	(2.5)	0	(0.0)	
Stage							0.593
III	26	(26.0)	21	(25.9)	5	(26.3)	
IV	62	(62.0)	49	(60.5)	13	(68.4)	
Recurrence	12	(12.0)	11	(13.6)	1	(5.3)	
Performance Status Score							0.13
0	23	(23.0)	14	(17.2)	9	(47.4)	
1	57	(57.0)	47	(58.0)	10	(52.6)	
2	12	(12.0)	12	(14.8)	0	(0.0)	
3	8	(8.0)	8	(9.9)	0	(0.0)	
PD-L1 status							0.003^a^
50–74%	37	(37.0)	30	(37.0)	7	(36.8)	
75–89%	26	(26.0)	22	(27.2)	4	(21.1)	
≥90%	30	(30.0)	27	(33.3)	3	(15.8)	
≥50% (details are unknown)	7	(7.0)	2	(2.5)	5	(26.3)	
Regimens							
CDDP+PEM + pembrolizumab	—		—		2	(10.5)	
CBDCA+PEM + pembrolizumab	—		—		4	(21.1)	
CBDCA+nab-PTX + pembrolizumab	—		—		13	(68.4)	

CBDCA, carboplatin; CDDP, cisplatin; nab-PTX, nab-paclitaxel; PEM, pemetrexed; PTX, paclitaxel.

^a^Including cases whose PD-L1 status details are unknown.

Twenty patients (24.7%) with a PS score of 2–3 were enrolled in the MONO group. There were no significant differences in the patient characteristics, except for PD-L1 expression, between the MONO and COMB groups. In the COMB group, 2 (10.5%) were treated with cisplatin + pemetrexed + pembrolizumab, 4 (21.1%) with carboplatin + pemetrexed + pembrolizumab and 13 (68.4%) with carboplatin + nab-paclitaxel + pembrolizumab.

### Efficacy

**Figure 1:**
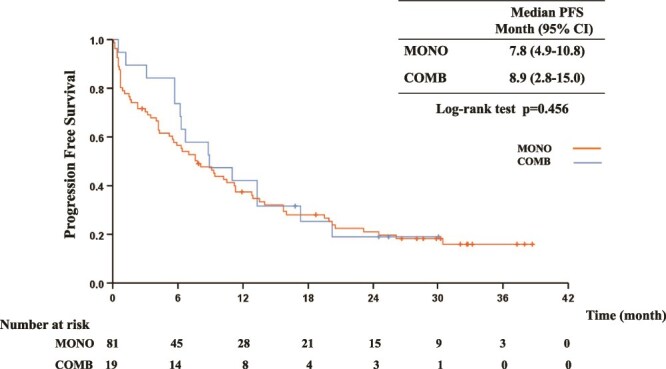
Kaplan–Meier curves for the PFS survival of elderly patients receiving pembrolizumab monotherapy (MONO) or pembrolizumab plus platinum-based chemotherapy (COMB). mo, month; PFS, progression-free survival.

**Figure 2:**
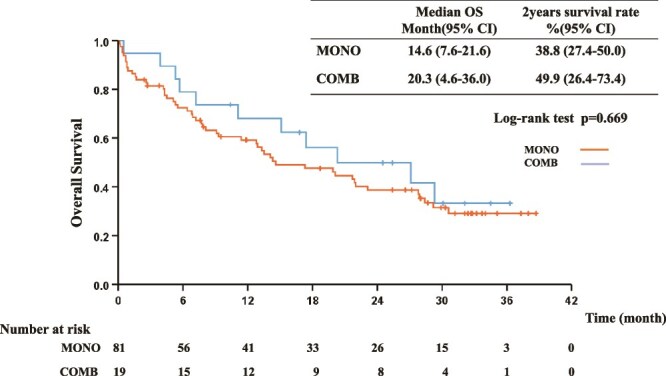
Kaplan–Meier curves for the OS of elderly patients receiving pembrolizumab monotherapy (MONO) or pembrolizumab plus platinum-based chemotherapy (COMB). mo, month; OS, overall survival.

The median PFS was 7.8 months (95% CI: 4.9–10.8) and 8.9 months (95% CI: 2.8–15.0) in the MONO and COMB groups, respectively [*P* = 0.456, HR 1.13 (0.64–1.99); Fig. 1]. The median OS was 14.6 months (95% CI: 7.6–21.6) in the MONO group and 20.3 months (95% CI: 4.6–36.0) in the COMB group [*P* = 0.669, HR 1.28 (0.67–2.46)]. The 2-year survival rates were 38.8% (95% CI: 27.4–50.0) and 49.9% (95% CI: 26.4–73.4) in the MONO and COMB groups, respectively (Fig. 2).

**Figure 3:**
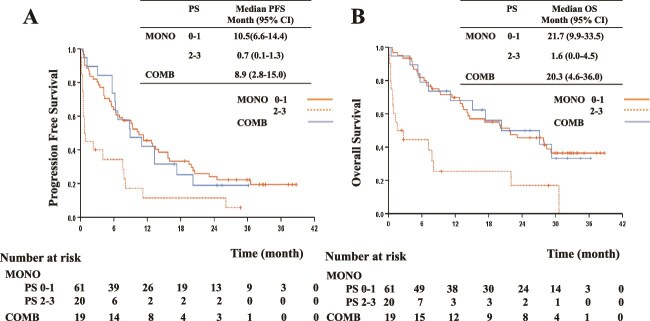
Kaplan–Meier curves for the PFS and OS of patients receiving pembrolizumab monotherapy (MONO) or pembrolizumab plus platinum-based chemotherapy (COMB) according to the ECOG PS. (A) PFS with a PS score of 0–1 or 2–3, (B) OS with a PS score of 0–1 or 2–3. ECOG: Eastern Cooperative Oncology Group; mo: month; OS, overall survival; PFS, progression-free survival; PS: performance status.

We also evaluated the PFS and OS in the PS subgroups. In the PS score 0–1 subgroup, there were no significant differences in PFS or OS between the MONO and COMB groups (PFS: 10.5 vs. 8.9 months, respectively; OS: 21.7 vs. 20.3 months, respectively) (Fig. 3A, B). However, in the PS score 2–3 subgroup, both PFS (0.7 months) and OS (1.6 months) tended to be shorter.

A confirmed objective response occurred in 33 patients (40.7%) treated with MONO [complete response (CR), *n* = 3; partial response (PR), *n* = 30] and 11 patients (57.9%) treated with COMB (CR, *n* = 0; PR, *n* = 11) (Table 2). Additionally, progressive disease rates were 28.4% (23/81) in the MONO group and 5.3% (1/19) in the COMB group (Table 2).

**Table 2:** Best tumor response to first line pembrolizumab monotherapy or pembrolizumab plus platinum-based chemotherapy

	No. (%)			
Tumor response	MONO		COMB	
	(*n* = 81)		(*n* = 19)	
ORR	33	(40.7)	11	(57.9)
DCR	53	(65.4)	16	(84.2)
Best overall response				
CR	3	(3.7)	0	(0.0)
PR	30	(37.0)	11	(57.9)
SD	20	(24.7)	5	(26.3)
PD	23	(28.4)	1	(5.3)
NE	5	(6.2)	2	(10.5)

COMB, pembrolizumab plus platinum-based chemotherapy; CR, complete response; DCR, disease control rate; MONO, pembrolizumab monotherapy; NE, not be evaluated; ORR, objective response rate; PD, progressive disease; PR, partial response; SD, stable disease

Among the PS score of 2–3 patients treated with MONO, the objective response rate was 15.0% (3 patients achieved PR), while 45.0% had progressive disease. All three patients who achieved PR had the PS score of 2. The two long-term survivors mentioned were both the PS score of 2 patients who had achieved PR. We observed no objective responses or long-term survival among the PS score of 3 patients.

### Safety and toxicity

At the data cutoff date, 93 patients (93%) discontinued treatment. Treatment discontinuation rates were 92.6% (75/81) and 94.7% (18/19) in the MONO and COMB groups, respectively. Disease progression was the most common reason for treatment discontinuation.

Twenty-four (29.6%) and five (26.3%) patients in the MONO and COMB groups, respectively, discontinued treatment due to AEs. There were no significant differences in AEs leading to discontinuation between the MONO and COMB groups. Pneumonitis was the most commonly reported AE, occurring in 10 (12.3%) patients in the MONO group and three patients (15.8%) in the COMB group. All cases of pneumonitis resulted in treatment discontinuation, including seven (8.6%) patients in the MONO group and two (10.5%) patients in the COMB group with grade 3 pneumonitis (Table 3). The frequently reported AEs in the COMB group were myelosuppression, including a decrease in the neutrophil count (15.8%) and platelet count (5.3%).

**Table 3:** Treatment-related adverse events grade ≥ 3 and leading to the discontinuation of all treatment

	No. (%)			
	MONO (*n* = 81)		COMB (*n* = 19)	
AEs	≥Grade 3	DISCON	≥Grade 3	DISCON
Total	24 (29.6)	24 (29.6)	7 (36.8)	5 (26.3)
Pneumonitis	7 (8.6)	10 (12.3)	2 (10.5)	3 (15.8)
Adrenal insufficiency	3 (3.7)	1 (1.2)		
Thromboembolic event	2 (2.5)	3 (3.7)	—	1 (5.3)
Enterocolitis	2 (2.5)	1 (1.2)	—	—
Rash	1 (1.2)	2 (2.5)	1 (5.3)	—
Neutrophil count decreased	1 (1.2)	1 (1.2)	3 (15.8)	—
Cholangitis	1 (1.2)	1 (1.2)	—	—
Fever	1 (1.2)	1 (1.2)	—	—
Pericarditis	1 (1.2)	1 (1.2)	—	—
Pharyngeal ulcer	1 (1.2)	1 (1.2)	—	—
Polymyalgia rheumatica	1 (1.2)	1 (1.2)	—	—
Hepatic dysfunction	1 (1.2)	—	—	—
Hyponatremia	1 (1.2)	—	—	—
Vasculitis	1 (1.2)	—	—	—
Heart failure	—	1 (1.2)	—	1 (5.3)
Diarrhea	—	1 (1.2)	—	—
Platelet count decreased	—	—	1 (5.3)	—

AE, adverse event; COMB, pembrolizumab plus platinum-based chemotherapy; DISCON, discontinuation; MONO, pembrolizumab monotherapy.

In the PS score of 2–3 subgroups, grade 3 AEs occurred in 3 (15.0%) patients and 3 (15.0%) patients discontinued treatment due to AEs.

### Analyses of PFS and OS according to various factors

Multivariate analyses using Cox proportional hazards modeling were performed to assess the correlations between various factors, PFS and OS. A PS score of 0–1 was identified as a good factor for PFS and OS (Table 4).

**Table 4:** Multivariate analysis of progression-free survival and overall survival

			All patients (*n* = 100)					
			Multivariate analysis of progression-free survival			Multivariate analysis of overall survival		
Variable			HR	95% CI	*P*	HR	95% CI	*P*
Age (y.o.)	<80 vs. ≥ 80		0.74	(0.46–1.17)	0.196	0.79	(0.47–1.34)	0.381
Sex	Male vs. female		1.58	(0.78–3.19)	0.201	2.22	(0.94–5.20)	0.068
PS	0–1 vs. ≥2		0.31	(0.17–0.57)	<0.001	0.24	(0.12–0.46)	<0.001
Smoking	Yes vs. no		0.82	(0.41–1.66)	0.588	0.67	(0.29–1.52)	0.333
Stage	III/IV vs. Rec		1.07	(0.50–2.30)	0.858	1.55	(0.71–3.40)	0.272
Pathology	Non-SQ vs. SQ		0.94	(0.57–1.54)	0.798	0.94	(0.59–1.78)	0.940
PD-L1 status	≥90 vs. 50–89%		0.67	(0.40–1.14)	0.140	0.62	(0.34–1.12)	0.113
Treatment	COMB vs. MONO		1.06	(0.56–2.00)	0.856	0.98	(0.47–2.03)	0.947

CI, confidence interval; COMB, combination therapy; HR, hazard ratio; MONO, monotherapy; PS, performance status; Rec, recurrence; SQ, squamous cell carcinoma; y.o., years old.

## Discussion

In our analyses, there was a trend toward choosing MONO for elderly patients with advanced-stage NSCLC and high PD-L1 expression. No significant differences were observed between the MONO and COMB groups in terms of PFS, OS and response rates. Patients in the COMB group tended to exhibit a longer PFS and OS than those in the MONO group. The incidences of grade 3 or higher AEs and AEs leading to treatment discontinuation were similar in both groups. All patients with a PS score of 2–3 received MONO, and their prognosis was poor even with high PD-L1 expression.

The investigators chose either MONO or COMB in elderly patients; however, the proportion of MONO was higher in the elderly population than in the overall analysis (55%) (6,7). We assume that COMB is more likely to increase the incidence and severity of AEs in elderly patients who might not be able to tolerate them. Notably, COMB was never selected for elderly patients with a PS score of 2–3.

A pooled analysis of the KEYNOTE-010, KEYNOTE-024 and KEYNOTE-042 studies for MONO included 132 patients aged 75 years and older with high PD-L1 expression. Pembrolizumab as the first-line therapy also improved the OS among elderly patients with high PD-L1 expression compared with chemotherapy [HR: 0.41 (95% CI: 0.23–0.73)] (16). In the elderly subgroup analysis of KEYNOTE-407, regardless of PD-L1 expression, COMB tended to result in a better PFS and OS compared to chemotherapy [HR: 0.61 (0.30–1.26) and HR: 0.85 (0.31–2.31), respectively]. In contrast, the elderly subgroup analysis of KEYNOTE-189, also regardless of PD-L1 expression, showed that COMB tended to have a worse PFS and OS compared to chemotherapy [HR: 1.73 (0.77–3.90) and HR: 2.09 (0.84–5.23), respectively] (9). However, these analyses did not specifically focus on patients aged 75 years and older with high PD-L1 expression levels.

In the patient subgroups with high PD-L1 expression and aged 75 years and older, a pooled analysis of phase III clinical trials reported a median PFS of 7.2 months in the MONO group and 11.8 months in the COMB group. The median OS were 18.9 months in the MONO group and not reached in the COMB group (17). Recent real-world data suggests that MONO is generally preferred for patients aged 75 years and older with high PD-L1 expression (13,18). In our analysis, PFS and OS tended to be better with COMB than with MONO; however, none of these differences were statistically significant. This finding suggests that COMB may not achieve a significant clinical benefit in this population.

In the multivariate analysis, PS was the only factor that significantly influenced both PFS and OS. We hypothesized that this may be because the COMB study did not include patients with a PS score of 2–3. When comparing PFS and OS for patients with a PS score of 0–1 only, the Kaplan–Meier curves for MONO and COMB were almost comparable. In contrast, patients with a PS score of 2–3 who received MONO had shorter PFS and OS times. Therefore, MONO did not demonstrate a favorable therapeutic effect in elderly patients with NSCLC, high PD-L1 expression and a poor PS score. The ECOG PS score is the most important factor in treatment selection; however, it is not the only factor. In patients with the lower the albumin levels, early increases in PK levels of pembrolizumab are less likely, potentially diminishing efficacy even when PD-L1 ≥ 50% (19). The geriatric assessment (GA) may be necessary when considering treatment options. Recently, several guidelines recommended the GA for elderly patients (20–22). Elderly patients often have coexisting conditions and tend to experience AEs. Therefore, although we did not evaluate the albumin levels and the GA in this study, we speculate that it may be necessary to consider treatment options regarding the future treatment selection for the elderly population.

Compared with previous reports, the incidence of immune-related AEs did not differ between the MONO and COMB groups (1–3). In elderly patients, pembrolizumab was associated with fewer AEs overall (68.5 vs. 94.3%) and fewer grade ≥ 3 AEs (24.2 vs. 61.0%) compared to chemotherapy (16). In this analysis, there was no significant difference in AE frequency between the MONO and COMB groups. However, few patients in the COMB group experienced myelosuppression, which may be attributed to chemotherapy components. In fact, in our study, 4.3% of patients under the age of 75 years developed myelosuppression, compared to 15.8% of patients aged 75 years and older. Nevertheless, there was no observed increase in treatment discontinuation due to AEs in the COMB group compared to the MONO group. Therefore, as there is no difference in therapeutic efficacy between MONO and COMB and AEs are more frequent with COMB, we consider MONO to be the preferred treatment option for elderly patients with high PD-L1 expression and a good PS score. However, COMB is rarely discontinued owing to AEs, and some patients may benefit from the early prevention of disease progression. In patients with a poor PS score, high PD-L1 expression does not necessarily indicate a good prognosis, and MONO may not be effective.

This study has some limitations. First, this was a retrospective study and included a subgroup analysis, which may have introduced bias in the regimen selection. However, we present data stratified by the PS score and compare the treatment efficacies of MONO and COMB. Second, the sample size was relatively small; however, we were able to demonstrate realistic treatment choices in clinical practice. Finally, not all AEs were recorded.

In conclusion, MONO is the preferred treatment for elderly patients with advanced-stage NSCLC and high PD-L1 expression. While the COMB group did not demonstrate a statistically significant benefit compared with the MONO group, it is noteworthy that some patients may benefit from COMB depending on patient selection. MONO may not be an effective or feasible treatment for patients with a PS score of 2–3, even in those with high PD-L1 expression.

## Supplementary Material

S1_jjcoj_hyae168

## Data Availability

The data that support the findings of this study are available from the corresponding author upon reasonable request.
