# Supplementary material for: Real-world data of first-line treatment with pembrolizumab for NSCLC with high PD-L1 expression in elderly patients: a subgroup analysis of HOT/NJLCG2001
Source: Jpn J Clin Oncol. 2024 Dec 4;55(3):253–60. doi: 10.1093/jjco/hyae168 (PMC11882503; doi:10.1093/jjco/hyae168)
Supplement: S1_jjcoj_hyae168 [file s1_jjcoj_hyae168.pptx]

## Slide 1
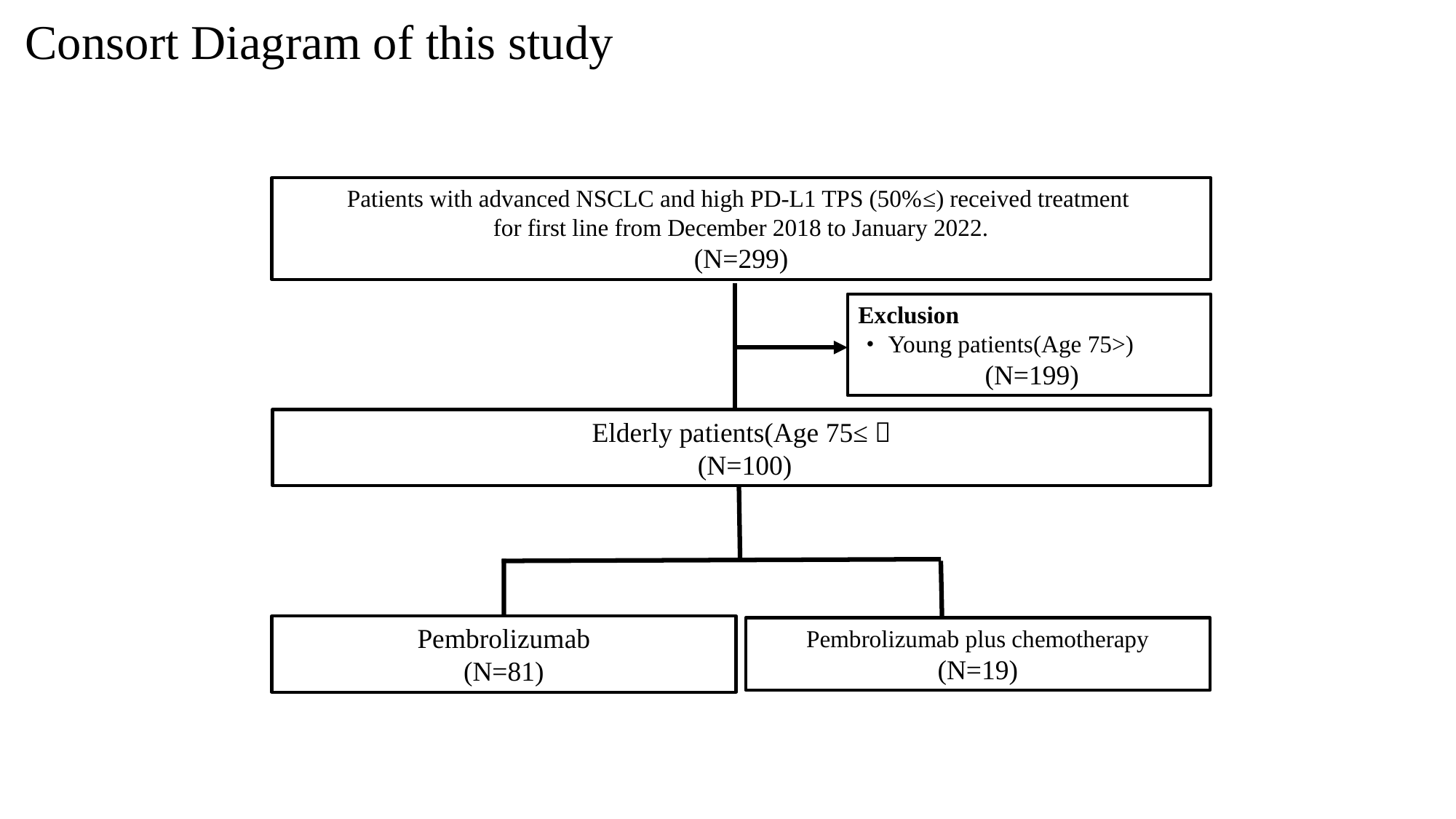

Consort Diagram of this study
Patients with advanced NSCLC and high PD-L1 TPS (50%≤) received treatment
for first line from December 2018 to January 2022.
(N=299)
Exclusion
・Young patients(Age 75>)
 (N=199)
Elderly patients(Age 75≤）
 (N=100)
Pembrolizumab
(N=81)
Pembrolizumab plus chemotherapy
(N=19)
